# The evolution of the Sin1 gene product, a little known protein implicated in stress responses and type I interferon signaling in vertebrates

**DOI:** 10.1186/1471-2148-5-13

**Published:** 2005-02-07

**Authors:** Shu-Zong Wang, R Michael Roberts

**Affiliations:** 1 Veterinary Pathobiology, University of Missouri, Columbia, USA; 2 Biochemistry, University of Missouri, Columbia, USA; 3 Animal Sciences, University of Missouri, Columbia, USA; 4 Center for Developmental Biology, University of Texas Southwestern, Medical Center, Dallas USA

## Abstract

**Background:**

In yeast, birds and mammals, the SAPK-interacting protein 1 (Sin1) gene product has been implicated as a component of the stress-activated protein kinase (SAPK) signal transduction pathway. Recently, Sin1 has also been shown to interact with the carboxyl terminal end of the cytoplasmic domain of the ovine type I interferon receptor subunit 2 (IFNAR2). However, the function of Sin1 remains unknown. Since SAPK pathways are ancient and the IFN system is confined to vertebrates, the organization of the Sin1 gene and the sequences of the Sin1 protein have been compared across a wide taxonomic range of species.

**Results:**

Sin1 is represented, apparently as a single gene, in all metazoan species and fungi but is not detectable in protozoa, prokaryotes, or plants. Sin1 is highly conserved in vertebrates (79–99% identity at amino acid level), which possess an interferon system, suggesting that it has been subjected to powerful evolutionary constraint that has limited its diversification.

Sin1 possesses at least two unique sequences in its IFNAR2-interacting region that are not represented in insects and other invertebrates. Sequence alignment between vertebrates and insects revealed five Sin1 strongly conserved domains (SCDs I-V), but an analysis of any of these domains failed to identify known functional protein motifs. SCD III, which is approximately 129 amino acids in length, is particularly highly conserved and is present in all the species examined, suggesting a conserved function from fungi to mammals. The coding region of the vertebrate Sin1 gene encompasses 11 exon and 10 introns, while in *C. elegans *the gene consists of 10 exons and 9 introns organized distinctly from those of vertebrates. In yeast and insects, Sin1 is intronless.

**Conclusions:**

The study reveals the phylogeny of a little studied gene which has recently been implicated in two important signal transduction pathways, one ancient (stress response), one relatively new (interferon signaling).

## Background

Sin1 was originally described as a human protein that modulated Ras function in *Saccharomyces cerevisiae *[1]. Strains of yeast that expressed the constitutively activated *RAS2*^Val 19 ^mutation had elevated levels of cyclic AMP, impaired growth control and were acutely sensitive to heat shock. This phenotype was reversed when the yeast strain was transfected with a cDNA (clone JC310) that encoded a then unknown protein. Although the authors suggested that the inferred interaction between the JC310 product and RAS might be fortuitous, they favored the possibility that that the unknown protein either was a true inhibitor of RAS or that it was a RAS target protein, which when over-expressed, had a protective action. A *S. cerevisiae *protein encoded by the AVO1 gene showed distant similarity the human JC310 product [2,3].

Approximately eight years after the identification of JC310 it was again identified, on this occasion in a yeast two-hybrid screen of a *Schizosaccharomyces pombe *cDNA library as a 665 amino acid protein that bound via polypeptide sequences in its C-terminal 244 amino acids to the Sty1/Spc1, stress activated MAP kinase (SAPK) [2]. A fission yeast strain lacking the Sin1 gene was sterile, sensitive to multiple types of stress, including heat shock, and had delayed cell cycles compared to a parental strain. Sin1 acted downstream of activated Sty1/Spc1 and appeared to be necessary for normal function of the transcription factor Atf1, a homolog of human ATF2. Wilkinson et al. [2] found that an apparent full length homolog of Sin1 from chicken allowed the heat sensitive strain of *S. pombe *to grow at 37°C, albeit very poorly. Moreover, fusion of the first 486 amino acids of yeast Sin1 (which does not restore growth) with the C-terminal 182 amino acids of the chicken Sin1 sequence protected against heat shock. Together, these data showed that Sin1 functions as a component of the stress-activated Sty1/Spc1 MAP kinase pathway in *S. pombe *and that a functional homolog of Sin1 exists in vertebrates.

No further information concerning Sin1 appeared since the paper of Wilkinson et al. [2] until our discovery that the ovine (ov) Sin1 associated via its C-terminus to the cytoplasmic domain of IFNAR2, a subunit of the type I IFN receptor [4], and Schroder et al. [5] described transcripts for Sin1 in human tissue and provided an analysis of the human gene. The latter study confirmed that Sin1 was relatively well conserved across Metazoa and fungi (Ascomyctes and Basidiomycetes) and was also represented in amoebae, but not in other protozoan species.

Ovine Sin1, which is 88% identical in sequence to chicken Sin1, can be co-immunoprecipated with the IFN receptor subunit IFNAR2 and shows a similar subcellular distribution to the receptor protein when co-expressed in mammalian cells [4]. Although ovSin1 was identified from a cDNA present in ovine endometrium and was initially considered to have a role in reproduction associated with the action of IFN-τ on the uterus during early pregnancy in the sheep, it became clear that the Sin1 gene was expressed in tissues other than endometrium and might have a general role in the action of type 1 IFN. In particular, it seemed possible that Sin1 might link the action of IFN to the stress activated SAPK signal transduction pathways. Such a linkage has been inferred from earlier studies in which early activation of p38 MAPK had been noted following exposure of a variety of cell lines to IFN-α, -β, or -τ [6-12].

Although the SAPK pathway is itself ancient and is found in all the species in which the Sin1 gene exists, the IFN system of receptors and ligands is restricted to vertebrates. We reasoned, therefore, that an analysis of Sin1 gene sequences might not only provide insight into the function of Sin1, but indicate how the protein evolved to interact with IFNAR2. The fact that the Sin1 gene appears to be expressed ubiquitously, that it is highly conserved across a wide range of taxa, and that it is a likely participant in several important signaling pathways, makes it an intriguing candidate for a functional/evolutionary analysis.

## Results

### Conservation of the Sin1 gene from yeast to mammals

A combination of searching methods was employed to locate Sin1 genes in available cDNA and genome data bases (Table 1). Sin1 sequences were found in two yeast species (*Schizosaccharomyces pombe *and *Saccharomyces cerevisiae*), the red bread mold (*Neurospora crassa*) and a number of other fungal species (not shown here), *Caenorhabditis elegans*, a mosquito species (*Anopheles gambiae*), fruit fly (*D. melanogaster*), frog (*Xenopus laevis*), two fish species (*Fugu rubripes *and *Danio rerio*), chicken (*Gallus gallus*), mouse (*Mus musculus*), rat (*Rattus norvegicus*), human (*Homo sapiens*), sheep (*Ovis aries*), cattle (*Bos taurus*), and pig (*Sus scrofa*) (Table 1). No apparent ortholog could be detected in the plant *Arabidopsis thaliana*. Nor could sequences corresponding to Sin1 be found in protozoa other than amoebae and prokaryotic species.

**Table 1:** Sin1 genes and their GenBank accession numbers

**Organism**	**GenBank Accession No.**	**Comments**
Yeast (*Saccharomyces cerevisiae*	NP_014563	Blastp the yeast protein database with fission yeast Sin1 protein.
Yeast (*Schizosaccharomyces pombe*	NP_594703	Wilkinson et al. 1999.
Red bread mold (*Neurospora crassa*	XP_322410	Blastp protein databases with budding yeast Sin1 protein.
Worm (*Caenorhabditis elegans*)	NM_064195	Blastp the worm protein database with ovSin1 protein.
Fly (*Drosophila melanogaster*)	AE003814	Blastp the fly protein database with ovSin1 protein.
Mosquito (*Anopheles gambiae*)	XM_319576	Blastp the mosquito protein database with ovSin1 protein.
Fish (*Fugu rubripes*)	N.A.	Blastp the fugu protein database with ovSin1 protein.
Frog (*Xenopus lavis*)	BC043789	Search EST databases with chicken Sin1 cDNA.
Chicken (*Gallus gallus*)	AF153127	Wilkinson et al. 1999.
Mouse (*Mus musculus*)	BQ713136, BF781677, BU152256	Search mouse EST and genome databases with sheep Sin1 cDNA
Rat (*Rattus norvegicus*)	CK476507, BE127132, BF553331, BU759329, AW141364	Search EST and genome databases with sheep Sin1 cDNA.
Pig (*Sus scrofa*)	CF791532, CF178115, BP459453, CF177341	Search EST databases with sheep Sin1 cDNA.
Cattle (*Bos taurus*)	BF230134, AV603930, CB433957, BM480500	Search EST databases with sheep Sin1 cDNA.
Sheep (*Ovis aries*)	AY547378	Wang oberts, 2004
Human (*Homo sapiens*)	NM_024117, BC002326	Search human EST and genome database with sheep Sin1 cDNA

"Comments" briefly describe the methods used to obtain the sequences.

The marked dissimilarity in inferred amino acid sequence between Sin1 from vertebrates and *C. elegans *(25% identity, Table 2), between the two yeast species (29% identity, Table 2; see Additional file: 1) and between *S. pombe *and *N. crassa *(28% identity, Table 2, see Additional file: 2) in the approximately 500 aa of overlap suggests that even if homologs existed in plants and prokaryotes they would likely be overlooked by the search methods employed.

**Table 2:** Pairwise comparisons of Sin1 cDNA and amino acid sequences from various species

	**S. pomb e **(665 aa)	**N. crassa **(798 aa)	**C. elegans **(684 aa)	**D. melanogaster **(569 aa)	**A. gambiae **(548 aa)	**F. Rubripes **(530 aa)	**X. lavis **(520 aa)	**G. gallus **(522 aa)	**M. musculus **(522 aa)	**R. norvegicus **(522 aa)	**O. aries **(522 aa)	**B. Taurus **(522 aa)	**S. scrofa **(522 aa)	**H. sapiens **(522 aa)
**S. pombe **(665 aa)	-	28.2	34.1*	28.8*	35.6*	21.7	28.2	21.1	24.6	26.1	25.5	25.4	24.8	25.3
**N. crassa **(798 aa)	NA	-	32.6*	28.3*	30.7*	22.6	31.9*	20.3	36.2*	20.5	20.9	20.9	20.9	20.5
**C. elegans **(684 aa)	NA	NA	-	27.5*	29.4*	22.3	22.6	21.8	23.0	23.0	23.5	23.2	22.8	23.2
**D. melanogaster **(569 aa)	NA	NA	NA	-	46.0	31.8	34.5	32.9	35.2	35.3	31.1	31.7	32.0	31.9
**A. gambiae **(548 aa)	NA	NA	NA	NA	-	34.9	33.4	35.3	33.8	34.0	33.2	33.0	33.2	33.4
**F. rubripes **(530 aa)	NA	NA	NA	NA	NA	-	80.0	82.9	78.7	79.2	79.8	80.2	79.8	80.4
**X. lavis **(520 aa)	NA	NA	NA	NA	NA	71.8	-	88.5	84.4	85.0	84.6	85.2	85.2	85.6
**G. gallus **(522 aa)	NA	NA	NA	NA	NA	74.7	78.0	-	88.0	88.3	88.3	89.3	89.3	90.0
**M. musculus **(522 aa)	NA	NA	NA	NA	NA	73.6	76.1	80.9	-	99.2	91.7	96.9	97.3	96.9
**R. norvegicus **(522 aa)	NA	NA	NA	NA	NA	73.6	76.3	80.5	96.2	-	91.3	96.9	96.9	96.9
**O. aries **(522 aa)	NA	NA	NA	NA	NA	73.3	75.3	81.7	96.0	96.0	-	98.7	97.9	98.1
**B. taurus **(522 aa)	NA	NA	NA	NA	NA	73.4	75.6	81.7	91.9	91.8	98.2	-	98.9	99.0
**S. scrofa **(522 aa)	NA	NA	NA	NA	NA	73.1	75.8	82.3	92.5	92.2	94.7	95.7	-	98.7
**H. sapiens **(522 aa)	NA	NA	NA	NA	NA	73.9	75.9	82.8	92.4	92.5	94.1	94.9	95.7	-

Notes: 1. Numbers in the upper-right half above the diagonal are identity percentages for amino acid sequences.

2. Numbers in the lower-left half below the diagonal are identity percentage for DNA sequences.

3. Numbers below the species names are the lengths of the Sin1 protein.

4. NA, Not applicable, i.e. no significant similarity was found.

5. Astericks, significant similarity occurs only in one region of the protein. For details, see the notes below:

S. pombe-C. elegans: significant similarity occurs in one region (170 aa: 252–391).

S. pombe- D. melanogaster : significant similarity occurs in one region (120 aa: 278–407).

S. pombe- A. gambiae: significant similarity occurs in one region (94 aa: 282–375).

N.crassa-C. elegans: significant similarity occurs in one region (90 aa: 376–465).

N.crassa-D. melanogaster: significant similarity occurs in one region (58 aa: 407–464).

N.crassa-A. gambiae: significant similarity occurs in one region (207 aa: 250–456).

N.crassa-Xenopus: significant similarity occurs in one region (127 aa: 338–464).

N.crassa-M. musculus: significant similarity occurs in one region (127 aa: 338–464).

C. elegans-D. melanogaster: significant similarity occurs in one region (169 aa: 198–366).

C. elegans-A. gambiae: significant similarity occurs in one region (178aa: 198–375).

6. Sequences and their GenBank accession numbers are: *O. aries *(AY547378), *B. taurus *(BF230134, AV603930, CB433957, BM480500), *H. sapiens *(NM_024117, BC002326), *S. scrofa *(CF791532, CF178115, BP459453, CF177341), *M. muscus *(BQ713136, BF781677, BU152256), *R. norvegicus *(CK476507, BE127132, BF553331, BU759329, AW141364), *G. gallus *(AF153127), *X. laevis *(BC043789), *F. rubripes *(Sequence accessible at ), *D. melanogaster *(AE003814), *A. gambiae *(XM_319576); *S. pombe *(AL136521, NP_594703, CAB66311); *N. crassa *(XP_322410).

**Figure 1:**
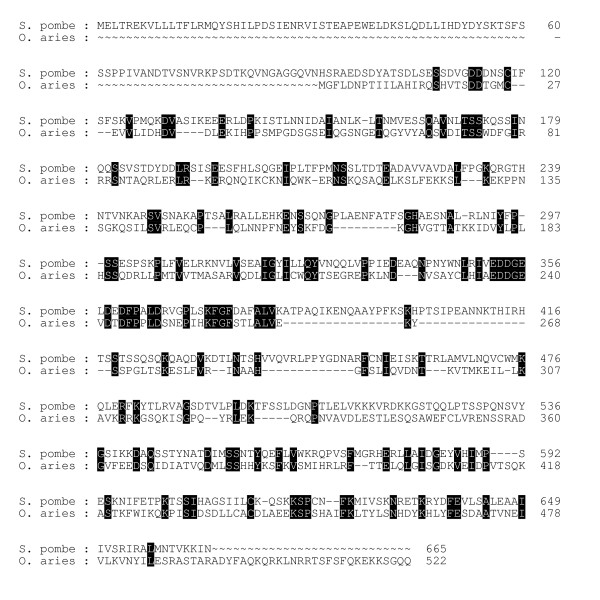
**Alignment of Sin1 proteins from the fission yeast and sheep. **The GAP program was used to align the two sequences. Black shading shows identical residues. Abbreviations: S. pombe, *Schizosaccharomyces pombe *(fission yeast. GenBank accession No. AL136521). O. aries, *Ovis aries *(sheep. GenBank accession No. AY547378).

**Figure 2:**
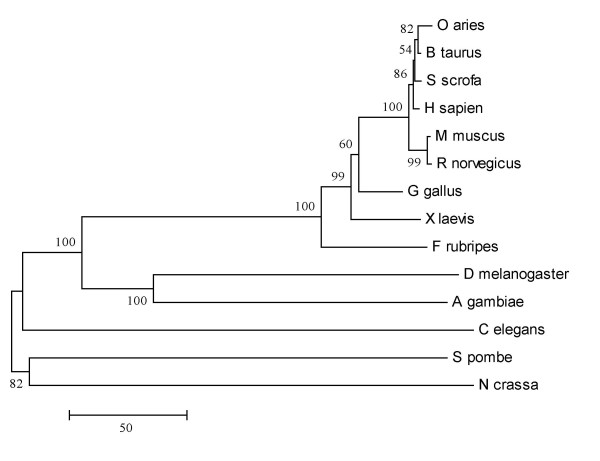
**A phylogenetic tree for Sin1 primary sequences from various species. **Sin1 polypeptide sequences were aligned by the program ClustalW, and the alignment output used by the program MEGA to generate a neighbor joining phylogenetic tree for the regions of alignment. GeneBank accession numbers for Sin1 sequences are listed in Table 1. Numbers beside branch points indicate the confidence levels for the relationship of the paired sequences as determined by bootstrap statistical analysis (1000 replicates). The lengths of the arms represent the extent of amino acid differences between the paired sequences, with the scale bar equivalent to 50 residues.

Sin1 from the yeast species, *S. cerevisiae *and *S. pombe *which consist of 1172 aa and 665 aa, respectively, and also from the red bread mold, *N. crassa *(798 aa) are much longer than Sin1 from vertebrate and insect species, which are ~520 aa long. The regions of similarity among these three fungal proteins are confined entirely to the carboxyl termini of these molecules, although several gaps have to be introduced to align them. No similarities are detectable in the amino terminal extensions, which, in the case of *S. cerevisiae*, is 370 aa long. It is the carboxyl regions of the fungal proteins that can also be aligned with the Sin1 sequences from *C. elegans*, insects, and vertebrate species, including *Ovis aries*, the sheep (Fig. 1).

A phylogenetic tree reconstructed from an alignment of amino acid sequences of Sin1 is shown in Fig. 2. As anticipated, the sequences from the three fungi, *C. elegans*, the two insect species, and vertebrate species fell into distinct branches of the tree. The sequences for the mammalian species were tightly clustered, with identities ranging from 99% (humans and cattle) to 91.3% (sheep and rat) (Table 2). All the vertebrate cDNA encoded polypeptides of 522 aa (Table 2).

**Figure 3:**
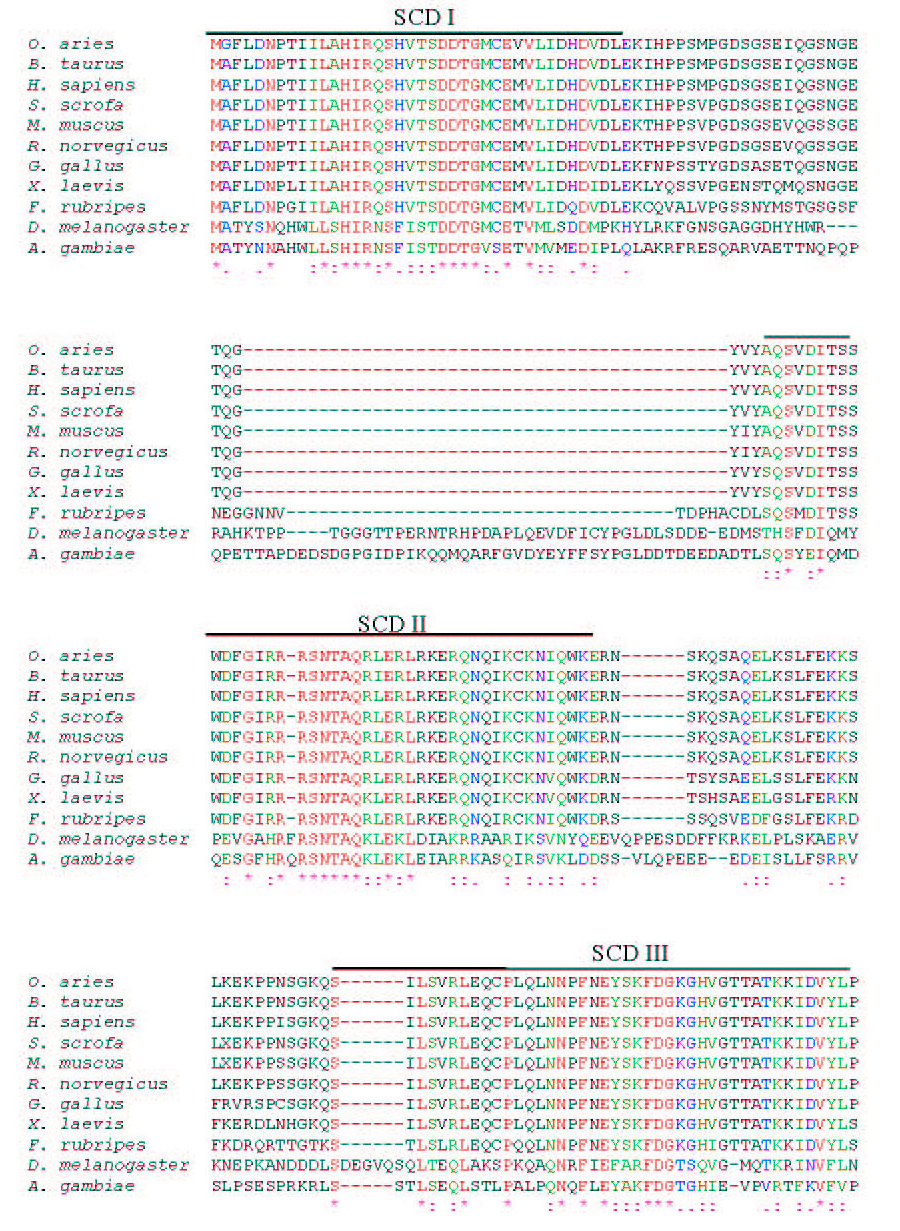
**The alignment of Sin1 polypeptide sequences from insects and vertebrates. **The ClustalW program was used to align all the protein sequences. Symbols (*, :, and .) show residues that are either identical(*), strongly similar (:), or weakly similar (.), respectively. Five Sin1 conserved domains (SCD) are highlighted as SCD I-V. The GenBank accession numbers for the sequences are: *O. aries *(AY547378), *B. taurus *(BF230134, AV603930, CB433957, BM480500), *H. sapiens *(NM_024117, BC002326), *S. scrofa *(CF791532, CF178115, BP459453, CF177341), *M. musculus *(BQ713136, BF781677, BU152256), *R. norvegicus *(CK476507, BE127132, BF553331, BU759329, AW141364), *G. gallus *(AF153127), *X. laevis *(BC043789), *F. rubripes *, *D. melanogaster *(AE003814), *A. gambiae *(XM_319576).

**Figure 4:**
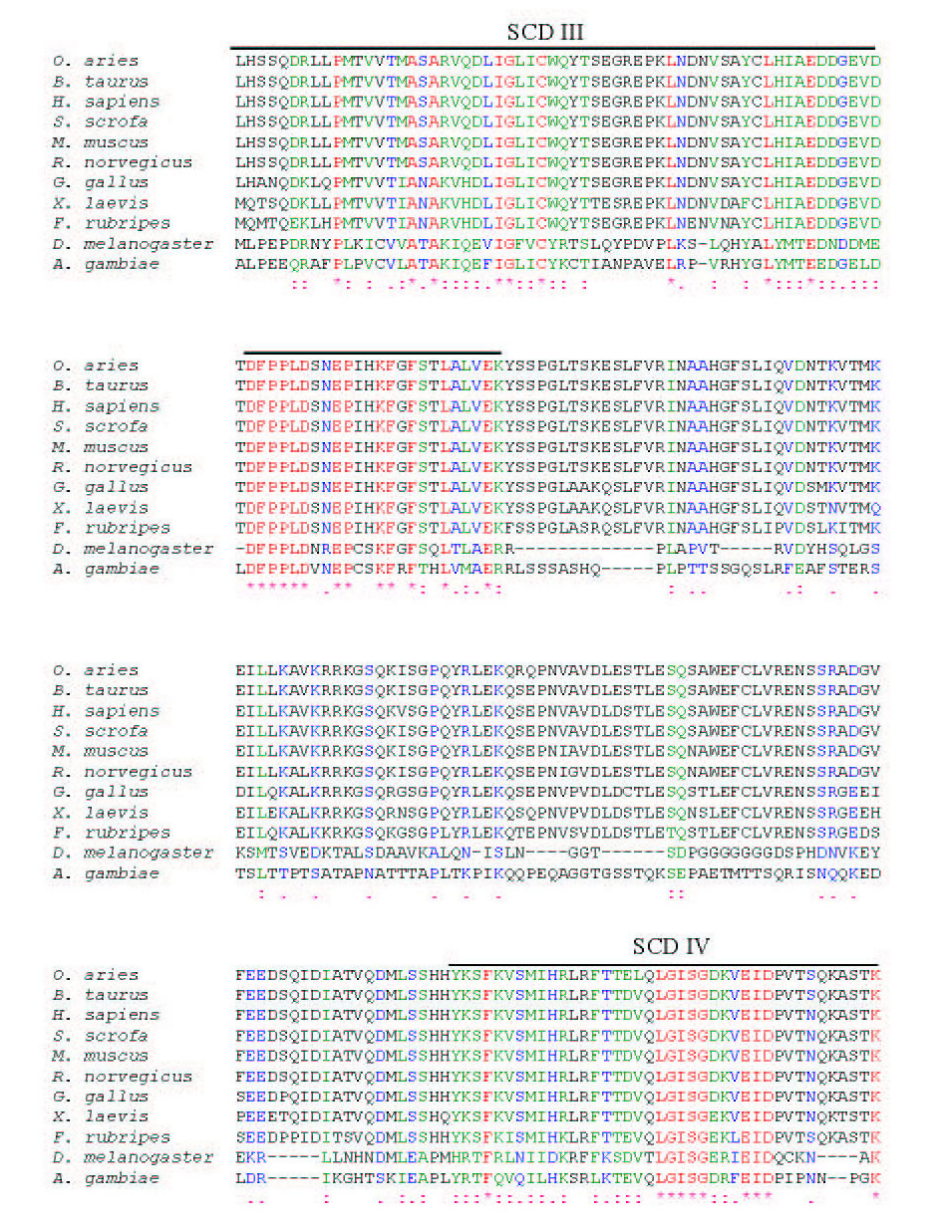
**The alignment of Sin1 polypeptide sequences from insects and vertebrates. **The ClustalW program was used to align all the protein sequences. Symbols (*, :, and .) show residues that are either identical(*), strongly similar (:), or weakly similar (.), respectively. Five Sin1 conserved domains (SCD) are highlighted as SCD I-V. The GenBank accession numbers for the sequences are: *O. aries *(AY547378), *B. taurus *(BF230134, AV603930, CB433957, BM480500), *H. sapiens *(NM_024117, BC002326), *S. scrofa *(CF791532, CF178115, BP459453, CF177341), *M. musculus *(BQ713136, BF781677, BU152256), *R. norvegicus *(CK476507, BE127132, BF553331, BU759329, AW141364), *G. gallus *(AF153127), *X. laevis *(BC043789), *F. rubripes *, *D. melanogaster *(AE003814), *A. gambiae *(XM_319576).

**Figure 5:**
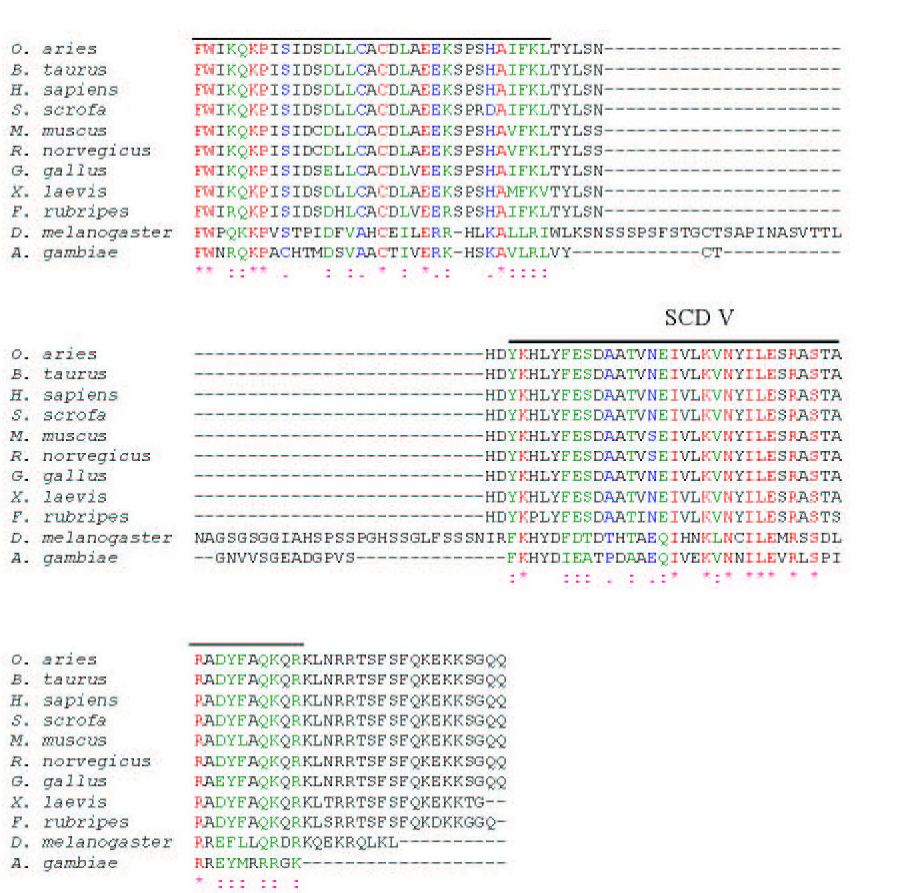
**The alignment of Sin1 polypeptide sequences from insects and vertebrates. **The ClustalW program was used to align all the protein sequences. Symbols (*, :, and .) show residues that are either identical(*), strongly similar (:), or weakly similar (.), respectively. Five Sin1 conserved domains (SCD) are highlighted as SCD I-V. The GenBank accession numbers for the sequences are: *O. aries *(AY547378), *B. taurus *(BF230134, AV603930, CB433957, BM480500), *H. sapiens *(NM_024117, BC002326), *S. scrofa *(CF791532, CF178115, BP459453, CF177341), *M. musculus *(BQ713136, BF781677, BU152256), *R. norvegicus *(CK476507, BE127132, BF553331, BU759329, AW141364), *G. gallus *(AF153127), *X. laevis *(BC043789), *F. rubripes *, *D. melanogaster *(AE003814), *A. gambiae *(XM_319576).

**Figure 6:**
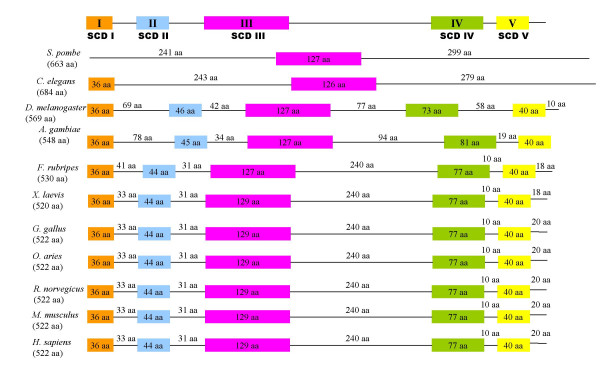
**The five highly conserved domains of Sin1 proteins. **Sin1 primary sequences from various species were aligned by using the ClustalW program, and the five most conserved domains identified from the alignment in Fig. 3 and the sequences listed in Table 1. Conserved domains are shown as boxes with remaining regions as solid lines. SCD, ***S***in1 ***c***onserved ***d***omain. Numbers beneath the species names are the lengths of the Sin1 proteins. Values in the boxes are the number of amino acid residues within a conserved domain. Numbers on the lines reflect the lengths of that region.

There is considerable conservation of Sin1 from mammals to birds (~90%), amphibians (~85%), and fish (~80%) (Table 2). The insect sequences are rather longer than the ones from vertebrates, and several gaps have to be introduced to provide alignments (Fig. 3, 4, 5). Nevertheless, the insect amino acid sequences are approximately 33% identical to those of the mammals (Table 2). Five blocks of sequence (SCD I-V) are significantly more conserved than others when two insects, a fish, an amphibian, a bird and several mammals are compared (Figs. 3, 4, 5 &6). Three of these regions are located towards the N-terminus and two additional regions towards the C-terminus. The most diverse region is located centrally.

**Figure 7:**
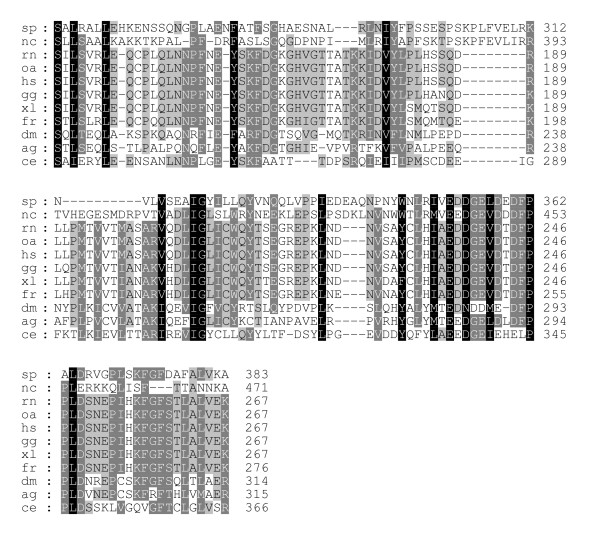
**Alignment of Sin1 conserved domain III from various species. **Sequences have been aligned by using the GCG PILEUP and GeneDoc programs. Degree of conservation is illustrated by intensity of shading (black, complete identity; light gray with black letters, complete identity across some but not all species; dark gray with white letters, high conservation but with conservative differences). The GenBank accession numbers for the sequences are: mm, *M. musculus *(BQ713136, BF781677, BU152256); rn, *R. norvegicus *(CK476507, BE127132, BF553331, BU759329, AW141364); bt, *B. taurus *(BF230134, AV603930, CB433957, BM480500); oa, *O. aries *(AY547378); ss, *S. scrofa *(CF791532, CF178115, BP459453, CF177341); hs, *H. sapiens *(NM_024117, BC002326); gg, *G. gallus *(AF153127); xl, *X. laevis *(BC043789); fr, *F. rubripes*); dm, *D. melanogaster *(AE003814); ag, *A. gambiae *(XM_319576); ce, *Caenorhabditis elegans *(NM_064195); sp, *Schizosaccharomyces pombe *(AL136521, NP_594703, CAB66311); nc, *Neurospora crassa *(XP_322410).

The region of the greatest identity between these divergent insect and vertebrate sequences is an acidic region placed in conserved SCD III (Fig. 3, 4, 5). In mammals, this region is completely conserved and corresponds to residues L232-K267 (LHIAEDDGEVDTDFPPLDSNEPIHKFGFSTLALVEK; Figs. 3, 4, 5; Fig. 7). However, an analysis of this sequence reveals no known functional motifs and no strong similarity to sequences represented in other known proteins. Schroder et al. [5] have also noted this conserved sequence in their analyses of Sin1 sequences and have named it CRIM for ***c***onserved ***r***egion ***i***n the ***m***iddle.

Sin1 from *C. elegans *retains the highly conserved 36 amino acid SCD I and the 127 amino acid Domain III (Fig. 6 &7). SCD III is also retained in the fission yeast and the red bread mold.

Vertebrates possess several unique sequences not present in insects and yeast, and, therefore, potentially implicated in the IFN signal transduction pathway including a carboxyl terminal region (KLSRRTSFSFQKDKK) immediately following the end of SCD V.

### Functional motifs in the Sin1 primary sequence

When the ovine Sin1 sequence is scanned for functional motifs [13], the structure appears unusually barren. Two weak bipartite nuclear localization signals (NLS) [14] can be detected. One (residues 82–98, RRSNTAQRLERLRKERQ) is present in the SCDII domain, and the other (residues 503–519, RKLNRRTSFSFQKEKKS) is almost at the C-terminus within conserved domain V (Fig. 3). Nevertheless, data from the subcellular localization experiment showed that Sin1 is excluded from the nucleus when transfected in COS1 or L929 cells [4], suggesting these NLS are probably not functional.

There are numerous motifs that are recognized as potential but weak sites for phosphorylation by either casein kinase II (CK2), protein kinase C, or protein kinase A (data not shown). None of the 17 CK2 sites, the 12 protein kinase C, or the 5 protein kinase A sites present in the ovine Sin1 primary sequence are conserved from mammals to fission yeast, although many are retained across all vertebrates. A weak site for myristylation (ovine residues 170–175, GTTATK; Figs. 1 &3, 4, 5), and hence for membrane association, is retained in all the vertebrate species examined, but is absent in insects and yeast. In absence of any data on the functional significance of these sites, they will not be discussed further.

### Gene structure of Sin1 from various species

**Figure 8:**
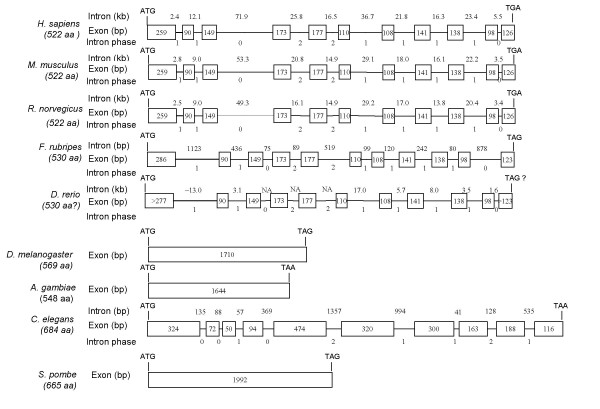
**A comparison of the Sin1 gene structure across species. **The gene structure for all species was retrieved from the genome database of the species by using the BLASTn program to analyze the open reading frame of each Sin1 cDNA sequence. Only the regions of the gene containing the open reading frame are shown in the diagram. All sequences begin with start codons and end with stop codons. The numbers under species names are the protein length.

The genomic sequence encompassing the transcribed region of the gene could be retrieved from the genome data bases for *S. pombe*, *S. cerevisiae*, *C. elegans*, *D. melanogaster*, *A. gambiae*, *F. rubripes*, *R. norvegicus*, *M. Musculus*, *H. sapiens *[15,16]. Sin1 exists as a single copy gene in all these species. For example, the human Sin1 gene is located on chromosome 9 (9q34.11-9q34.12) (data not shown) with the transcribed region composed of 11 exons and 10 introns and spanning a region of about 240 kb (Fig. 8). Exon 7 is spliced out of the shorter form of Sin1 [4,5]. The lack of exon 7 does not cause a frame shift because the intron phases of the two introns on both sides of exon 7 are identical (data not shown). Schroder et al. [5] have also demonstrated or predicted other minor splice variants for Sin1 in the human. The 11 exons account for only 0.9% of the gene sequence. It is, of course, unclear how many additional exons and introns are associated with the 5' UTR beyond the transcription start site(s), whose location has not been determined.

Currently, the sheep and bovine genome sequences are not available, but it is likely that the Sin1 gene organization will be similar to that in the human. The current comparative synteny maps between human, sheep and cattle [17-19] predict that the Sin1 gene is located on sheep chromosome 3 (3p1.7-3p2.6) and bovine chromosome 11 (11q2.3-11q2.8), respectively.

A comparative map for all the genes is shown in Fig. 8. In fission yeast and insects, the Sin1 gene consists of a single exon. In worm, fish, rat, mice, and human, Sin1 has multiple exons. The exon/intron pattern, consisting of 11 exons, is observed in all vertebrates, including the two fish species (Fig. 8). It is noteworthy that although the genomic sequences of sheep and cattle are not available, the exon/intron pattern of their Sin1 genes is similar to that of other vertebrates based on the comparison between sheep or cattle Sin1 cDNA and human genomic sequence of Sin1 (data not shown). The lengths of these 11 exons are also remarkably conserved and fall within the normal range (50–200bp for most internal exons) (International Human Genome Sequencing Consortium, 2001). As expected, the sizes of the introns differ across species, and some are extremely long. Intron sizes generally decrease in the order human > mouse > rat > fish (Fig. 8). As expected, intron sizes were quite similar between rodents and human.

The Sin1 gene from *C. elegans *is organized quite differently from that in mammals. It consists of 10 exons interrupted by nine relatively short introns. The region of the *C. elegans *gene that contains regions of similarity with the mammalian protein sequences consists of exon 1 (SCD I) and exon 5 (SCD III). As noted above and in Figure 8, the Sin1 gene from insects and *S. pombe *is comprised of only a single exon.

## Discussion

Sin1 is a little studied gene product of unclear function found in species ranging from mammals to fungi. Although the *S. pombe *gene product is longer than that of mammals, with an extension at its N-terminus, human Sin1 can rescue the stress sensitivity noted in the phenotype of a *S. pombe *strain that expressed a constitutively active form of RAS, indicating that function, as well as structure, has been conserved over hundreds of millions of years.

Two facts should be considered when attempting to infer a role for Sin1 in vertebrates. The first, as discussed in the Background, is the known ability of type 1 IFN to activate MAPK/SAPK in mammalian cells. The second is the proven involvement of Sin1 in the yeast SAPK (Sty1/Spc1) pathway and its involvement in controlling transcription of stress-activated genes [2]. The present analysis was conducted in an attempt to gain more detailed information about Sin1 function from a phylogenetic analysis and comparison of Sin1 genes and gene products in different taxonomic groups.

The Sin1 gene is remarkably divergent in both length and sequence identity within the fungi *S. pombe*, *S. cerevisiae*, *and N. crassa*, emphasizing the evolutionary distance between these three species. The regions of similarity are confined to the ~600 amino acid C-terminal regions of the three sequences (data not shown), and it is this region that is also conserved in insects and vertebrates (see Additional file: 1 &2). This diversity in structure within the fungi is probably reflected in divergence of function. AVO1, the apparent Sin1 ortholog of *S. cerevisiae*, forms a membrane-associated complex with TOR2 and other protein components (AVO2, AVO3 and LST8), which control cell growth in response to nutrients [3,32]. Cells with deletion of AVO1 are unable to organize their actin cytoskeleton [3]. In contrast, the Sin1 ortholog of *S. pombe *is involved in a stress response signaling pathway by interacting with Sty1 [2]. A cross-species comparison of all the Sin1 sequences available, indicates five regions of greatest conservation, only one of which, a ~127 amino acid central region (SCD III), was easily defined in all taxa (Figs. 3, 4, 5 &6). Even this region is poorly conserved in the budding yeast, *S. cerevisiae*, although certain landmark amino acids are retained (data not shown). Interestingly, Sin1 from insects and vertebrates, despite having only about 35% identity, are of similar length and possess the five regions of high identity. Conceivably, the SCD III domain is functionally essential in all the species, while SCDs I, II, IV, and V have evolved conserved function within the Metazoa. A not unreasonable assumption is that that Sin1 plays an evolutionarily conserved role in SAPK signaling across a broad range of taxa, including all metazoan and fungal species [5] but has assumed an additional function in vertebrates in mediating crosstalk with the IFN-signal transduction pathway.

In vertebrates Sin1 falls into a class of highly conserved gene products. Its conservation is lower than that of two structural proteins, histone H3 and β-actin, but is comparable to that of CDK1 (Table 3). However, while CDK1 in yeast and insects retains considerable sequence identity with the vertebrate orthologs, much of the conservation of Sin1 is lost. It is tempting to speculate that Sin1 has been subjected to powerful evolutionary constraint that has limited its amino acid sequence divergence within vertebrates. It should be noted that our analyses cannot exclude the possibility that conservation of Sin1 among vertebrates reflects recent divergence of the sampled vertebrates relative to the other taxa examined. Once data become available, it will be instructive to compare Sin1 gene sequences from the invertebrate chordates (Tunicata and Cephalochordata) with those of the other metazoan taxa.

**Table 3:** Comparison across species of the amino acid sequence conservation of Sin1 with some other conserved genes

	**Yeast**	**Drosophila**	**Frog**	**Chicken**	**Mouse**	**Cattle**	**Human**
**Histone H3**	91.2%	98.5%	94.9%	95.6%	97.8%	98.5%	100%
**β-actin**	90.4%	97.9%	99.5%	100%	100%	98.1%	100%
**CDK1**	64.9%	71.7%	88.5%	93.3%	97.0%	98.7%	100%
**Sin1**	25.3%	31.9%	85.6%	90.0%	96.9%	99.0%	100%

Values for percentage identities were obtained by aligning amino acid sequences from various species with their human counterparts. CDK, cyclin-dependent kinase. The GenBank accession numbers for sequences are as follows. Human (*Homo sapiens*): histone H3 (AAH66884), β-actin (NP_001092), CDK1 (P06493), Sin1 (NM_024117, BC002326). Cattle (*Bos taurus*): histone H3 (P16105), β-actin (AAM98378), CDK1 (P48734), Sin1 (BF230134, AV603930, CB433957, BM480500). Mouse (*Mus musculus*): histone H3 (NP_062342), β-actin (NP_031419), CDK1 (NP_031685), Sin1 (BQ713136, BF781677, BU152256). Chicken (*Gallus gallus*): histone H3 (I50245), β-actin (NP_990849), CDK1 (P13863); Sin1 (AF153127). Frog (*Xenopus laevis*): histone H3 (P02302), β-actin (AAC27796), CDK1 (P35567, Sin1 (BC043789). Fly (*Drosophila melanogaster*): histone H3 (NP_724345), β-actin (NP_511052), CDK1 (NP_476797), Sin1 (AE003814). Yeast (*Schizosaccharomyces pombe*): histone H3 (NP_595567), β-actin (NP_595618), CDK1 (NP_595629), Sin1 (NP_014563).

Sin1 was shown to be associated with the cytoplasmic domain of IFNAR2, a subunit of the type I IFN receptor [4]. Since insects appear to lack genes for type I IFN and their receptors (R. M. Roberts, unpublished observations), whereas vertebrates utilize this system primarily as an anti-viral response [20-22], it should be theoretically possible to define a sequence *in silico *unique to vertebrates but clearly absent in both *D. melanogaster *and *A. gambiae *that might account for the association of Sin1 with IFNAR2. Sin1 binds to the carboxyl end of the cytoplasmic domain of IFNAR2 via its own carboxyl 114 amino acids [4]. At least two candidate sequences exist in that part of Sin1. One is the rather basic carboxyl terminus (aa 510–522), another a HDYKHLYFESDA (aa 458–469) sequence, both of which are absent in the insect proteins (Figs. 3, 4, 5). Whether these sequences are participants in the interaction of Sin1 with IFNAR2 in vertebrates has not been examined experimentally. Of course, it is quite possible that insect Sin1 can bind vertebrate IFNAR2 or that amino acid substitutions elsewhere in the carboxyl end of the vertebrate sequence have evolved to promote the interaction. These possibilities have also not been tested. In this regard, IFNAR2, with which Sin1 interacts, has evolved much more rapidly than Sin1 itself. The sequence of human IFNAR2, for example, shows only about 58% and 29% identity to those of ovine and chicken IFNAR2, respectively [21,23], while orthologs have yet to be defined for IFNAR2 in frogs and fish, even though these animals are believed to have a functional IFN system, which includes the production of Type I IFN and downstream components in response to double stranded RNA [20,22]. Interestingly, the only highly conserved continuous sequence of chick and mammalian IFNAR2 within the Sin1 binding region is an acidic region (aa 493–515; human IFNAR2 numbering) at the very carboxyl terminus of the molecule ([23]; R.M. Roberts, unpublished observations). It seems possible that this conserved sequence provides the scaffold for Sin1 binding.

As also observed by Schroder et al. [5], Sin1 is represented by a single gene in all species where it exists. In both insects and the two yeast species, the gene is intronless, while in *C. elegans *and in vertebrate species introns are present (Fig. 8). In budding yeast, only a small number (3.8%) of genes have introns [24], whereas in most other eukaryotes, including *Drosophila*, intronic sequences are a feature of the majority of genes and must be excised to produce a functional mRNA [25]. For *D. melanogaster*, for example, there is an average of 3 introns per gene [26]. These introns are short, averaging 240 bp in *Drosophila *[27]. Why the Sin1 genes are intronless in these species is unclear, but there is considerable evidence that retrotransposition occurs in yeast, *Drosophila *[28] and mammals [29]. In this process, reverse transcription of mRNA from a parental gene creates an intronless copy of the parental gene at a new position in the genome. If this mechanism created the Sin1 gene, a remnant or evolved version of the parental gene might be anticipated to exist, particularly if the transposition event occurred in recent evolutionary time [28]. It is unclear whether the intronless Sin1 gene in *Drosophila *resulted from such a retrotransposition event since there is not a detectable intronic copy elsewhere in the genome. The Sin1 gene from *C. elegans *has introns, but is organized very differently from that of vertebrates, where the intron/exon organization is highly conserved (Fig. 8).

Unfortunately, the function of Sin1 is unknown. Its structural conservation from vertebrates to yeast [30] and its expression in most, if not all tissues of mammals [4] suggest a central, if elusive, role in life processes.

## Conclusions

SAPK-interacting protein 1 (Sin1), a little-studied but widely expressed gene product, is encoded by a single gene in fungi, nematodes, insects, and all vertebrates analyzed and shows modest conservation of amino acid sequence that is consistent with some degree of conserved function in stress-activated signal transduction pathways. Sin1 is highly conserved in vertebrates where it has been implicated in linking interferon responses to the SAPK pathway.

## Methods

### Databases

Sin1 genomic sequences from human, mouse, rat, fruit fly, mosquito, *C. elegans*, *S. pombe*, and *S. cerevisiae*, were retrieved from at NCBI Genome databases [18]. Sin1 cDNA sequences from human, mouse, rat, cattle and pig, and other Sin1 ESTs were retrieved from GenBank EST database after BLASTn analysis at NCBI [18]. For fish Sin1 genomic sequences, the incomplete puffer fish (*Fugu rubripes*) and zebrafish (*Danio rerio*) genome databases at the Ensembl site [16] were used. The budding yeast (*Saccharomyces cerevisiae*) ORF (open reading frame) database [33] was used to retrieve budding yeast Sin1.

### Software programs used to analyze sequences

Pairwise global sequence alignment was performed by using either the BESTFIT or the GAP program from GCG (Madison, WI). Multiple global sequence alignment was performed by using either the PILEUP program (GCG, Madison, WI) and GeneDoc [34] or ClustalW program [35]. The phylogenetic tree for Sin1 was generated by using the ClustalW program and the MEGA program [36]. Motif search was performed by using the ScanProsite program [13].

### Methods for obtaining Sin1 sequences from various species

Fission yeast (*Schizosaccharomyces pombe*) and chicken (*Gallus gallus*): The two Sin1 sequences were published by Wilkinson et al. [2].

Budding yeast (*Saccharomyces cerevisiae*): The BLASTp program was used to search the budding yeast ORF database for any protein sequence that had significant similarity to the fission yeast Sin1 protein. The obtained budding yeast Sin1 protein sequence had a GenBank link where its cDNA was available. The cDNA sequence was used to analyze its genomic structure at the NCBI yeast genome site.

Red bread mold (*Neurospora crassa*): Sin1 protein was retrieved from the *Neurospora crossa *protein data base by searching (BLASTp) with the budding yeast Sin1 protein.

Worm (*Caenorhabditis elegans*): The Sin1 protein sequence was obtained from the *C. elegans *protein database by searching with ovine Sin1 protein. The cDNA sequence was then obtained from the GenBank link and used to determine the structure of the Sin1 gene.

Fly (*Drosophila melanogaster*): The fruit fly Sin1 protein sequence was retrieved from the *D. melanogaster *protein database as above. The cDNA sequence was obtained from the GenBank link. Unexpectedly, querying the Drosophila genomic sequence with the *C. elegans *Sin1 sequence and vice-versa failed to yield a match in either case.

Mosquito (*Anopheles gambiae*): The mosquito Sin1 protein sequence was retrieved from the *Anopheles gambiae str. PEST *protein database as above. The cDNA sequence was then obtained from the GenBank link.

Puffer fish (*Fugu rubripes*) and Zebrafish (*Danio rerio*): Both *Fugu rubripes *and *Danio rerio *genome databases, which are accessible at two websites, NCBI and ENSEMBL, were queried with Sin1 cDNA sequences from sheep, chicken, and frog. For both species, only the Ensembl site provided the complete genomic sequence. Although the *Fugu rubripes *genome sequence is incomplete, the exons of Sin1 cDNA can be retrieved and successfully assembled into the full length structure by alignment with other Sin1 cDNA and gene sequences. No GenBank entry was available for the *Fugu rubripes *Sin1 gene. When a similar method was used to retrieve the Zebrafish Sin1 cDNA sequence, the full length sequence could not be obtained because the region (~20 kb) covering one exon (exon 4) was incomplete. Therefore, the fish Sin1 protein sequence used here is from *Fugu rubripes*.

Frog (*Xenopus lavis*): The full-length cDNA sequence of Sin1 reported here was from African clawed frog, and was obtained by blasting the Xenopus EST database [37] with the chicken Sin1 sequence. The protein sequence was deduced from this cDNA sequence.

Mouse (*Mus musculus*): The mouse Sin1 cDNA sequence was obtained by editing several ESTs, after performing a BLASTn search of the *Mus Musculus *EST database with the ovine Sin1 cDNA sequence. Searching the mouse genome database with the mouse Sin1 cDNA coding region then allowed the gene, down stream of its transcription start site to be located and its structure to be inferred.

Rat (*Rattus norvegicus*): The rat Sin1 cDNA sequence was retrieved from several overlapping ESTs, which were obtained by searching the *Rattus norvegicus *EST database with the ovine Sin1 cDNA sequence. The coding region of the rat Sin1 cDNA was then used to search the rat genome database at the NCBI website for the genomic structure of the gene.

Cattle (*Bos taurus*): The full length bovine Sin1 cDNA sequence was obtained from overlapping ESTs, which were obtained by searching the NCBI EST database with the ovine Sin1 cDNA sequence.

Pig (*Sus scrofa*): The swine Sin1 cDNA sequence was obtained as above by searching the NCBI EST database with the ovine Sin1 cDNA sequence.

Human (*Homo sapiens*): The sequence published by Colicelli et al. [31] was confirmed by performing a BLASTn search on human EST data bases with the ovine Sin1 cDNA sequence. Since the previously published sequence was not full-length, an additional human Sin1 EST (GenBank Acc. No. BC002326) was used to assembly the full length Sin1 cDNA sequence. The location of the gene and its structure downstream of its transcription start site were determined by searching the full human genome database with the Sin1 open reading frame.

Sheep (*Ovis aries*): The sheep Sin1 cDNA sequence was cloned from a sheep endometrial cDNA library in a yeast two-hybrid screen [4]. GenBank accession numbers are summarized in Table 1.

## Authors' contributions

SW carried out the majority of the computational analyses under the direction of RMR, and wrote the first draft of the manuscript. RMR conceived of the study and participated in its design and coordination. All authors read and approved the final manuscript.

## Supplementary Material

Additional file 1 **Alignment of Sin1 proteins from the fission yeast and the budding yeast. **The Bestfit program was used to align the two sequences. Black shading shows identical residues. A conserved region (SCD III; see Fig. 4) is highlighted by a line above the sequence, and appears not so well conserved in the budding yeast as in other species. Abbreviations: S. pombe, *Schizosaccharomyces pombe *(fission yeast. GenBank accession No. AL136521). S. cerevisae, *Saccharomyces cerevisae *(budding yeast. GenBank accession No. NP_014563). Click here for file

Additional file 2 **Alignment of Sin1 proteins from the fission yeast and the red bread mold. **The Bestfit program was used to align the two sequences. Black shading shows identical residues. Abbreviations: S. pombe, *Schizosaccharomyces pombe *(fission yeast. GenBank accession No. AL136521). N. crassa, *Neurospora crassa *(red bread mold. GenBank accession No. XP_322410). Click here for file
