# Supplementary material for: The evolution of the Sin1 gene product, a little known protein implicated in stress responses and type I interferon signaling in vertebrates
Source: BMC Evol Biol. 2005 Feb 7;5:13. doi: 10.1186/1471-2148-5-13 (PMC549548; doi:10.1186/1471-2148-5-13)
Supplement: Additional file 1 — Alignment of Sin1 proteins from the fission yeast and the budding yeast. The Bestfit program was used to align the two sequences. Black shading shows identical residues. A conserved region (SCD III; see Fig. 4) is highlighted by a line above the sequence, and appears not so well conserved in the budding yeast as in other species. Abbreviations: S. pombe, Schizosaccharomyces pombe (fission yeast. GenBank accession No. AL136521). S. cerevisae, Saccharomyces cerevisae (budding yeast. GenBank accession No. NP_014563). [file 1471-2148-5-13-S1.doc]

S. pombe : GGQVNHSRAEDSDYATSDLSESSDVGDDDNSCIFSFSKVPMQKDVASIKEEERLDP 142
S. cerevisae: GSEYSEERYSNNDSSTMESGEMSLDSDMQTNTIPSHS-IPMSMQKYGIYHGD--DD 508

S. pombe : GGQVNHSRAEDSDYATSDLSESSDVGDDDNSCIFSFSKVPMQKDVASIKEEERLDP 142
S. cerevisae: GSEYSEERYSNNDSSTMESGEMSLDSDMQTNTIPSHS-IPMSMQKYGIYHGD--DD 508

S. pombe : KISTLNNIDAIANLKLTN----------MVESSQAVNLTSSKQSSINQQSSVSTDY 188
S. cerevisae: --STLNNVFDKAVLTMNSSRHPKERRDTVISGKEPTSLTSSNRKFSVSSNLTSTRS 562

S. pombe : DDLR-----SISEESFHLSQGEIP---LTFPMNSSLTDTEADAVVAVDALFPGKQR 236
S. cerevisae: PLLRGHGRTSSTASSEHMKAPKVSDSVLHRARKSTLTLKQDHSQPSVPSSVHKSSK 618

S. pombe : GTHNTVNKARSVSNAKAPTSALRALL--EHKENSSQNGPLAENFATFSGHAESN-- 288
S. cerevisae: EGNILIEKTTDYLVSKPKASQLSNMFNKKKKRTNTNSVDVLEYFSFVCGDKVPNYE 674

S. pombe : ALRLNIYFPSSESPSKPLF-VELRKNVLVSEAIGYILLQYVNQQLVPPIEDEA--- 340
S. cerevisae: SMGLEIYIQASKKYKRNSFTTKVRKSSTIFEVIGFALFLYSTEKKPDNFEEDGLTV 730

S. pombe : ---QNPNYWNLRIVEDDGE-LDEDFPALDRVGPLSKFGFDAFALVKATPAQIKENQ 392
S. cerevisae: EDISNPNNFSLKIVDEDGEPFEDNFGKLDRKSTIQSISDSEVVLCKVDDAEKSQNE 786

S. pombe : AAYPFKSKHPTSIPEAN----NKTHIRHTSSTSSQSQ--KQA---QDVKDTLNTSH 439
S. cerevisae: IETPLPFETGGGLMDASTLDANSSH-DTTDGTINQLSFYKPIIGNEDDIDKTNGSK 841

S. pombe : VVQVRLPPYGD-NARF--CNIEISKTTRLAMVLNQVCWMKQLERFKYTLRVAGSDT 492
S. cerevisae: IIDVTVYLYPNVNPKFNYTTISVLVTSHINDILVKYCKMKNMDPNEYALKVLGKNY 897

S. pombe : VLPLDKTFSSLDGNPTLELVKKK-VRD----------KK----------------- 520
S. cerevisae: ILDLNDTVLRLDGINKVELISKKDARELHLEKMKPDLKKPVLPTIQSNDLTPLTLE 953

S. pombe : -----------GSTQQLP-----TS------------------------------- 529
S. cerevisae: PLNSYLKADAGGAVAAIPENTKVTSKAKKISTKYKLGLAKQHSSSSVASGSVSTAG 1009

S. pombe : -------------SPQNSVYGSIK----KDAQSSTYNATDI----------MSSNT 558
S. cerevisae: GLANGNGFFKNKNSSKSSLHGTLQFHNINRSQSTMEHTPDTPNGVGDNFQDLFTGA 1065

S. pombe : YQEFLVWKRQPVSFMGRHERLLAIDGEYVHIMPSESK-NIFETPKTSSIHAGSIIL 613
S. cerevisae: YHKYKVWRRQQMSFINKHERTLAIDGDYIYIVPPEGRIHWHDNVKTKSLHISQVVL 1121

S. pombe : CKQSKKSPCNFKMIVSK--NRETKRYDFEVLSALEAAIIVSRIRALMNTVK 662
S. cerevisae: VKKSKRVPEHFKIFVRREGQDDIKRYYFEAVSGQECTEIVTRLQNLLSAYR 1172
