# Supplementary material for: The evolution of the Sin1 gene product, a little known protein implicated in stress responses and type I interferon signaling in vertebrates
Source: BMC Evol Biol. 2005 Feb 7;5:13. doi: 10.1186/1471-2148-5-13 (PMC549548; doi:10.1186/1471-2148-5-13)
Supplement: Additional file 2 — Alignment of Sin1 proteins from the fission yeast and the red bread mold. The Bestfit program was used to align the two sequences. Black shading shows identical residues. Abbreviations: S. pombe, Schizosaccharomyces pombe (fission yeast. GenBank accession No. AL136521). N. crassa, Neurospora crassa (red bread mold. GenBank accession No. XP_322410). [file 1471-2148-5-13-S2.doc]

S. pombe : SSSPPIVANDTVSNVRKPSDTKQVNGAGGQVNHSRAED-SDYATSDLSESSDVGDDDNSC 118
N. crassa : SGSSPIQS----SNLRQATTMSPPKPAVRRGSQSALETVKERARSDTVTSSEVSSDHEFD 205

S. pombe : IFSFSKVPMQKDVASIKEEERLDPKISTLNNI----DAIANLKLTNMVESSQAVNLTSSK 174
N. crassa : ASGFHRAREAAQAAAAR-AAKLSAKLNTDPSMGIKREASDLLEDEEDDDDSDASDISEAF 264

S. pombe : QSSINQQSSVSTDYDDLRSISEESFHLSQGEIPLTFPMNSSLTDTEADAVVAVDALFPGK 234
N. crassa : VESIDSASILDAIKNPMNASPQ---HQVVGTPPREYTRRSTM--IRKSVMPPPSSLLVGK 319

S. pombe : QRGTHNTVNKARSVSNAKAPTSALRALLEHKENSSQNGPLAENFATFSGHAESNALRLNI 294
N. crassa : -LPPPRPLSTIRPISVVQ-PKSLLSAALKAKKTKPAL-PF-DRFASLSGQGDPNPIMLRI 375

S. pombe : YFPSSESPSKPLFVELRKN----------VLVSEAIGYILLQYVNQQLVPPIEDEAQNPN 344
N. crassa : YAPFSKTPSKPFEVLIRRTVHEGESMDRPVTVADLIGLSLWRYNEEKLEPSLPSDKLNVN 435

S. pombe : YWNLRIVEDDGELDEDFPALDRVGPL-------SKFG--------FDAFALVKATPAQIK 389
N. crassa : WWTLRMVEEDGEVDDDFPPLERKKQLISFTTANNKAGRSRSNSKVYDIFALSKASEDEFE 495

S. pombe : ENQAAYP-FKSKH----------------PTSIPEANNK---------THIRHTSSTSSQ 423
N. crassa : ENQKLTPQFEQEQAGGSLEEEEEEDKDLTPRGTPRPDNSLLPAAEPRGNPLLNTTYRPGV 555

S. pombe : SQ--KQAQDVKDTLNTSHVVQ--VRLPPYGDN---ARFCNIEISKTTRLAMVLNQVCWMK 476
N. crassa : TMYADMPQPTQPTQSTSRGEKRLLRIHIHSSDVPAGQMITLDVTTETWLADVLDTACRKR 615

S. pombe : QLERFKYTLRVAGSDTVLPLDKTFSSLDGNPTLELVKKK-VRDKKGSTQQLP-TSSPQNS 534
N. crassa : QLDKANHVLKLPQSGIVVPLDRTVGSLNNVTELDLHRRRFATDGPLTMTGSPSSSSPRPP 675

S. pombe : VYGSIKKDAQSSTYNATDIM-----------------SSNTYQEFLVWKRQPVSFMGRHE 577
N. crassa : LFADNSSTWTTKSKKSRGIMGVHPLAKEILKQDELGIGATAVKKYTVWRKQPMRLLS--E 733

S. pombe : RLLAIDGEYVHIMPSE-SKNIFE--TPKTSSIHAGSIILCKQSKKSPCNFKM 626
N. crassa : KIFVIDGEYIHIMPSAGGKNPGEIVDGKATTVHFSNVVGCKVSRKHPNNFKL 785
